# Conformational variation of proteins at room temperature is not dominated by radiation damage

**DOI:** 10.1107/S1600577516017343

**Published:** 2017-01-01

**Authors:** Silvia Russi, Ana González, Lillian R. Kenner, Daniel A. Keedy, James S. Fraser, Henry van den Bedem

**Affiliations:** a Stanford Synchrotron Radiation Lightsource, SLAC National Accelerator Laboratory, Menlo Park, CA 94025, USA; b Department of Bioengineering and Therapeutic Sciences, UCSF, San Francisco, CA, USA; c Bioscience Department, SLAC National Accelerator Laboratory, Menlo Park, CA 94025, USA

**Keywords:** radiation damage, room temperature, cryo-temperature, conformational dynamics

## Abstract

Protein crystallography data collection at synchrotrons is routinely carried out at cryogenic temperatures to mitigate radiation damage. Although damage still takes place at 100 K and below, the immobilization of free radicals increases the lifetime of the crystals by approximately 100-fold. Recent studies have shown that flash-cooling decreases the heterogeneity of the conformational ensemble and can hide important functional mechanisms from observation. These discoveries have motivated increasing numbers of experiments to be carried out at room temperature. However, the trade-offs between increased risk of radiation damage and increased observation of alternative conformations at room temperature relative to cryogenic temperature have not been examined. A considerable amount of effort has previously been spent studying radiation damage at cryo-temperatures, but the relevance of these studies to room temperature diffraction is not well understood. Here, the effects of radiation damage on the conformational landscapes of three different proteins (*T. danielli* thaumatin, hen egg-white lysozyme and human cyclo­philin A) at room (278 K) and cryogenic (100 K) temperatures are investigated. Increasingly damaged datasets were collected at each temperature, up to a maximum dose of the order of 10^7^ Gy at 100 K and 10^5^ Gy at 278 K. Although it was not possible to discern a clear trend between damage and multiple conformations at either temperature, it was observed that disorder, monitored by *B*-factor-dependent crystallographic order parameters, increased with higher absorbed dose for the three proteins at 100 K. At 278 K, however, the total increase in this disorder was only statistically significant for thaumatin. A correlation between specific radiation damage affecting side chains and the amount of disorder was not observed. This analysis suggests that elevated conformational heterogeneity in crystal structures at room temperature is observed despite radiation damage, and not as a result thereof.

## Abstract 2

The conformational variation of three different proteins as a function of dose is examined at 278 and 100 K.

## 1. Introduction   

The vast majority of macromolecular crystallographic data are collected at cryogenic temperatures. This practice became widely adopted during the 1990s due to the realisation that cryo-cooling reduced radiation damage to the samples during the experiment (Haas & Rossmann, 1970 ▸; Hope, 1988 ▸). Concurrently, cryostats to maintain the sample in a stable cryo-temperature environment during data collection (Cosier & Glazer, 1986 ▸) and improved sample handling and cryo-cooling methods were developed (Garman & Schneider, 1997 ▸; Teng & Moffat, 1998 ▸; Rodgers, 1997 ▸; Garman, 1999 ▸; Pflugrath, 2004 ▸). The increase in the lifetime of the crystal at 100 K compared with room temperature is around two orders of magnitude (Nave & Garman, 2005 ▸), although some variation can occur depending on the sample, solvent content and dose rate (Warkentin & Thorne, 2010 ▸; Kmetko *et al.*, 2011 ▸; Southworth-Davies *et al.*, 2007 ▸; Leal *et al.*, 2013 ▸).

Despite the notable advantage of data collection at cryogenic temperatures for reducing radiation damage, there are several important considerations that can motivate collecting data at higher temperatures. The increase in mosaicity in cryo-cooled crystals can result in an excessive number of overlapped reflections in crystals with large unit cells; for example, with large macromolecular complexes and viruses (Gilbert *et al.*, 2003 ▸; Rossmann, 1999 ▸). Experiments at multiple temperatures can provide important information about protein conformational dynamics (Frauenfelder *et al.*, 1979 ▸; Tilton *et al.*, 1992 ▸; Saikrishnan *et al.*, 2005 ▸, Schmidt *et al.*, 2009 ▸; Weik & Colletier, 2010 ▸; Woldeyes *et al.*, 2014 ▸; Keedy *et al.*, 2015*a* ▸), and changes of temperature can be used to control chemical reactions and observe structural intermediates (Bourgeois & Royant, 2005 ▸; Colletier *et al.*, 2008 ▸). Furthermore, because it can induce structural changes and inhibit thermal motions, crystal cryo-cooling can obscure interpretation of the structure and potentially lead to erroneous conclusions regarding functional mechanisms (Deacon *et al.*, 1997 ▸; Scheidig *et al.*, 1999 ▸; Dunlop *et al.*, 2005 ▸; Fraser *et al.*, 2011 ▸; Keedy *et al.*, 2014 ▸; Fischer *et al.*, 2015 ▸). For these reasons, the practice of collecting data at room temperature is likely to continue and even grow in popularity.

X-ray free-electron lasers (XFELs) can avoid radiation damage by the ‘diffract-before-destruct’ approach (Neutze *et al.*, 2000 ▸) which was experimentally confirmed with doses up to 400 MGy (Chapman *et al.*, 2011 ▸, 2014 ▸; Boutet *et al.*, 2012 ▸). At synchrotron sources, where most experiments still take place, the problems posed by the shorter lifetime of the samples at room temperature can be mitigated by combining partial datasets from multiple samples or different parts of the same sample (Brodersen *et al.*, 2003 ▸; Axford *et al.*, 2012 ▸; Stellato *et al.*, 2014 ▸). However, radiation damage remains a particular concern for studies of molecular mechanisms and protein function because it could potentially reorganize the conformational distributions even at low doses. In cryo-cooled crystals, the specific effects of ionizing radiation can be explained by reduction by photoelectrons (reduction of metal centers, separation of di­sulfide bridges) or oxidation by electron holes (de­carboxyl­ation of glutamate and aspartate residues). Active-site acidic side chains have been shown to be particularly sensitive (Fioravanti *et al.*, 2007 ▸). Often, residues surrounding damaged sites reorganize (Burmeister, 2000 ▸; Ravelli & McSweeney, 2000 ▸; Weik *et al.*, 2000 ▸; Ravelli & Garman, 2006 ▸). At room temperature, the increased mobility of free radicals and protein domains results in quantitative and qualitative differences in the sensitivity of specific residues and domains to ionizing radiation. For example, Warkentin *et al.* (2012 ▸) found that solvent-exposed turns are more sensitive at room temperatures, while below 180 K increased sensitivity can be correlated with poor local packing. Juers & Weik (2011 ▸) also reported higher temperature factors (*B*-factors) for residues near solvent channels and more types of residues being damaged at 160 K than at 100 K.

Here, we have examined the effects of radiation damage on conformational distributions to determine its impact on studies where disorder and conformational heterogeneity play an important role in the activity of a macromolecule (van den Bedem, *et al.*, 2013 ▸; Woldeyes *et al.*, 2014 ▸; Fischer *et al.*, 2015 ▸; Bhabha *et al.*, 2015 ▸). A specific aim of the experiment described below is to determine whether radiation damage could play a role in the appearance or disappearance of biologically relevant alternative conformations and how it affects the inherent disorder in structures at 100 K and room temperature. We used *qFit* multi-conformer models (van den Bedem *et al.*, 2009 ▸; Keedy *et al.*, 2015*b* ▸) to interrogate differences in the conformational landscape of hen egg-white lysozyme (HEWL), *Thaumatococcus daniellii* thaumatin, and the human proline isomerase cyclo­philin A (CypA) at 100 K and 278 K after increasing amounts of irradiation in an X-ray beam. Crystals of these proteins diffract to high resolution, which makes it possible to fit alternate conformations. CypA is of special interest, since it is a well characterized dynamic enzyme where minor active-site side-chain conformations, absent in cryogenic electron-density maps, help explain the catalytic function of the enzyme, which isomerizes its substrate *via* correlated motions of the side chains (Fraser *et al.*, 2009 ▸). Comparison of radiation damage effects at cryogenic *versus* room temperature is complicated by the rapid decay of the crystals at room temperature. Thus, to be able to compare the structural conformational variety at roughly similar stages of decay, the crystals were subjected to very different doses at the two different temperatures, with the maximum dose at 100 K being about 30 MGy [the conventional limit for experiments at cryogenic temperatures (Owen *et al.*, 2006 ▸)], and about two orders of magnitude less at 278 K. Within each of those dose ranges we modelled and quantified alternative conformations as a function of accumulated dose.

## 2. Methods   

### 2.1. Crystal preparation   

HEWL lysozyme tetragonal (*P*4_3_2_1_2) crystals were grown by vapour diffusion from drops containing 40–60 mg ml^−1^ solution of protein and well solution (1.0 *M* sodium chloride in a 50 m*M* sodium acetate buffer pH = 4.5). The crystals used for data collection at 100 K were cryo-cooled in liquid nitro­gen after dipping them in mother liquor containing 25–30% ethyl­ene glycol.

*T. daniellii* thaumatin crystals belonging to space group *P*4_1_2_1_2 were grown by vapour diffusion, as described by Nanao *et al.* (2005 ▸), in 0.9 *M* Na/K tartrate, 100 m*M* HEPES pH = 7.4 and 15% glycerol, and cryo-protected for data collection at 100 K in mother liquor containing 30% glycerol.

Wild-type CypA was produced and crystallized in the space group *P*2_1_2_1_2_1_ as reported by Fraser *et al.* (2009 ▸). For data collection at 100 K, the crystals were cryo-protected in paratone oil.

All the crystals used for data collection at 278 K were mounted in cryo-mounts covered with low background MicroRT capillaries from MiTeGen containing well solution in the tip.

### 2.2. Data collection   

Data were collected following the same overall protocol for the three proteins (see details in Table S1 of the supporting information) with the beamline control and data collection software *Blu-Ice* (Soltis *et al.*, 2008 ▸; McPhillips *et al.*, 2002 ▸). Before data collection, estimates of the maximum dose for each crystal were obtained with *RADDOSE* v2 (Murray *et al.*, 2004 ▸; Paithankar & Garman, 2010 ▸) *via* *WebIce* (González *et al.*, 2008 ▸). The diffraction-weighted dose (DWD), a metric proposed by Zeldin *et al.* (2013*a* ▸) that takes into account the different volumes of the crystal being exposed during data collection and thus better representing the average damage received by the crystal, was afterwards recalculated using *RADDOSE-3D* (Zeldin *et al.*, 2013*b* ▸). The dose rate was around 30 kGy s^−1^. HEWL and thaumatin data were collected on beamline BL14-1, with a marMosaic325 detector at an energy of 10.2 keV. CypA data were collected on beamline BL7-1, equipped with an ADSC 315 detector using an energy of 11.27 keV. An Oxford CryoJet was used to maintain the temperature constant at 100 K or 278 K during data collection.

For data collection at 100 K, several consecutive datasets were collected from the same crystal until a total maximum accumulated dose around 30 MGy was reached. At this total dose, both a noticeable decrease in diffracted intensities and specific damage are expected (Owen *et al.*, 2006 ▸). The increase per data set of the DWD was 1.11 MGy for CypA, 1.86 MGy for thaumatin and 1.75 MGy for HEWL. At 278 K, the total maximum dose was limited to less than 0.3 MGy, almost two orders of magnitude less than for the data collection at 100 K. This dose did not permit the collection of full datasets at different stages of damage from a single-crystal to a sufficiently high resolution to model multiple conformations. We therefore used several crystals, collecting consecutive partial datasets from a small rotation wedge (typically a few degrees) that resulted in a DWD of 16 kGy per wedge for CypA, 23 kGy for thaumatin and 28 kGy for HEWL. These partial datasets were then combined to obtain fairly complete datasets. Decay of the diffraction was evidenced by a decrease in the resolution of the most damaged data.

### 2.3. Data processing   

All the data were autoindexed and processed with *XDS* (Kabsch, 2010 ▸). The CCP4 programs *POINTLESS* and *AIMLESS* (Winn *et al.*, 2011 ▸; Evans, 2006 ▸, 2011 ▸) were used to verify the symmetry and sort, scale and merge the reflections, both for single-crystal and multiple-crystal datasets. Amplitudes were calculated with *TRUNCATE* (Winn *et al.*, 2011 ▸). The data processing statistics are shown in Table 1 ▸. For each protein, the same initial models were refined independently against each dataset. Initial refinement was carried out with *REFMAC* (Murshudov *et al.*, 1997 ▸). The *qFit* 1.0 algorithm was used to examine and model alternative interpretations of the X-ray electron density map (van den Bedem *et al.*, 2009 ▸; Keedy *et al.*, 2015*b* ▸). Atomic position and occupancies of the *qFit* multiconformer models were further refined with *PHENIX* (Afonine *et al.*, 2012 ▸). For HEWL and thaumatin we used all the reflections available, relying on the refinement software to downweight the contribution of weak reflections; for CypA, however, the refinement results were not satisfactory when including the high-resolution reflections, perhaps because of the lower completeness in the highest resolution shells, and we applied a resolution cutoff at 1.7 Å, still sufficient to model double conformations. In all cases, the resulting models were inspected with the program *Coot* (Emsley *et al.*, 2010 ▸) and manual corrections were made where necessary. The refinement statistics are shown in Table S2.

### 2.4. Characterization of conformational redistribution   

The number of individual residues with alternative conformations present in the models were counted to evaluate the conformational ensembles as a function of temperature and radiation damage. To determine which conformations represented well differentiated positions of the side chain, as opposed to oscillations around the same rotamer or energy well, the average root-mean-square difference (RMSD) between the non-hydrogen atoms of alternate conformations was calculated with *LSQKAB* (Kabsch, 1976 ▸). An average difference of 1.0 Å was used as the lower cutoff value.

### 2.5. Characterization of conformational dynamics   

We examined the effects of radiation damage on conformational dynamics using the *B*-factor-dependent, multi-conformer crystallographic order parameters *S* ^2^ proposed by Fenwick *et al.* (2014 ▸). These order parameters include both harmonic contributions, encoded by the temperature factors, and non-harmonic contributions, encoded by occupancies and displacements in coordinates around a given bond vector. The crystallographic order parameters correlate well with NMR-determined order parameters and provide a meaningful quantification of the degree of order in the structure. The analysis was applied to the bond most closely associated with the χ1 side-chain dihedral angle, using Cβ—*X*β (where *X* = C or O) for most amino acids, Cα—Cβ for Ala, and Cα—Hα for Gly (Keedy *et al.*, 2015*a* ▸). Because *S* ^2^ is a measure of order, 1 − *S* ^2^ was used as a measure of disorder.

The average disorder for all residues in each structure was calculated. In particular, this analysis includes the CypA active-site residues known to be involved in correlated, functional motions in the room-temperature models.

### 2.6. Site-specific characterization of radiation damage   

To detect the sites most affected by radiation damage, the *F*_o__*n*_ − *F*_o__1_ difference maps were examined using the σ-weighted coefficients from the lowest and highest dose datasets. Peaks in the map were searched and sorted with *PEAKMAX* (Winn *et al.*, 2011 ▸). The maps were visually inspected with *Coot*.

## 3. Results   

Table 2 ▸ shows the number of residues with multiple conformations for each dataset. Side-chain alternative conformations are more abundant at 278 K than at 100 K for thaumatin and CypA, which is consistent with previous observations (Fraser *et al.*, 2011 ▸; van den Bedem *et al.*, 2013 ▸), but not for HEWL. There was no evidence found of cryoprotectant molecules binding to side chains with different conformations at 100 K. The number of multiple conformations seen even for the most damaged models indicates that conformational heterogeneity can be observed without interference from radiation damage. There is a decreasing trend for the total number of conformations as a function of dose for all three proteins at 278 K, but these trends are not statistically significant. By contrast, there is essentially no trend for number of conformations as a function of dose at 100 K for any of the three proteins.

**Figure 1:**
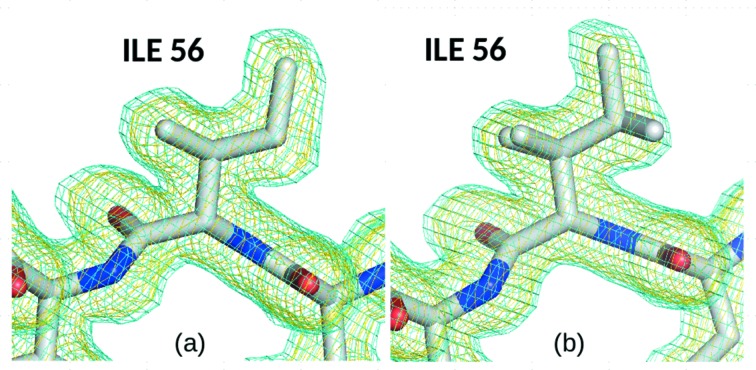
Example of buried isoleucine (I56 in CypA at 278 K) in a 2*F*_o_ − *F*_c_ map contoured at 1.0σ and 0.3σ. At the lowest absorbed dose (*a*) only one rotamer is modelled. At a higher dose (*b*) an alternate rotamer occupying the same density is fitted without visible changes in the map.

Many of the alternate conformations in the structures are unaffected by increasing damage. However, for each data set a small number of conformers vanish or appear in a non-systematic manner. These changes appear to be incidental and take place in sites where there is ambiguity in the interpretation of the electron density. They fall into one of these three types:

(*a*) Alternate conformations clustered around a single rotamer.

(*b*) Side chains of solvent exposed residues, or residues with high *B*-factors. In these cases the electron density for the minor conformers is not very well defined.

(*c*) Rotamers enabled by alternate main-chain conformations (Davis *et al.*, 2006 ▸) in buried, ordered residues, where the alternate side-chain conformations fit reasonably well in the electron density for both rotamers (see example in Fig. 1 ▸).

**Figure 2:**
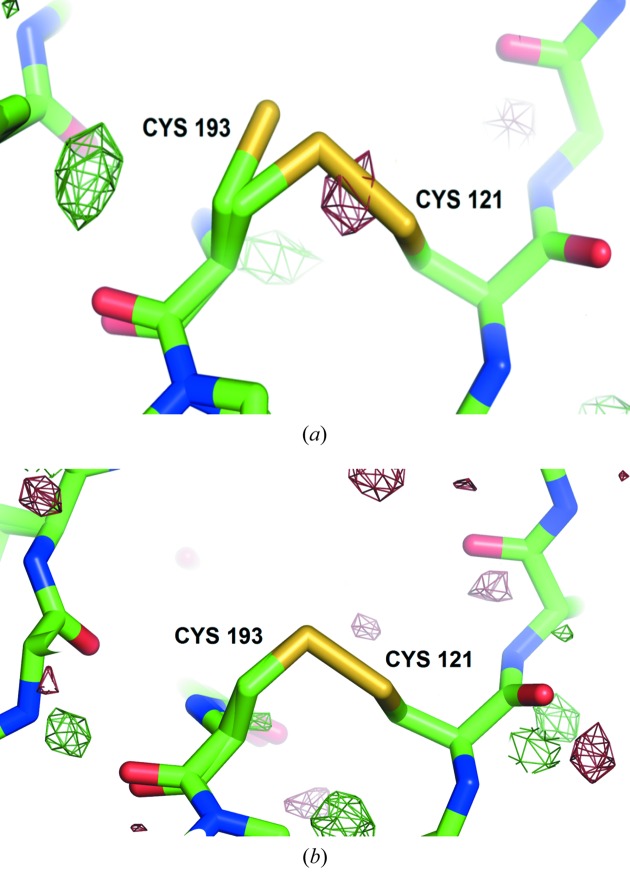
Di­sulfide bond C121—C193 in thaumatin at the highest absorbed dose. At 100 K (*a*), one of the cysteines could be modelled in an alternate conformation, while negative density, contoured at 2.5σ between the bonded sulfurs, indicates that photoreduction has taken place. At 278 K (*b*), the negative density is not visible at the same contour level and the map does not support modelling of a second conformation.

In general, movement of side chains was not associated with a strong peak in the *F*_o__*n*_ − *F*_o__1_ electron density map based on the lowest and highest dose datasets (see Table S3). We also examined the sites with large peaks in the *F*_o__*n*_ − *F*_o__1_ maps to look for conformational variation. At 278 K, large differences appear for carboxyl O atoms and amine nitro­gens in the main chain, and also cysteines, phenyl­alanines and the polar side-chains asparagines and threonines. At 100 K, damage was concentrated mainly on cysteines, me­thio­nines, aspartates, glutamates and main-chain carboxyl O atoms. The only residues where a distinct alternate conformation could be clearly attributed to radiation damage were some of the cysteines in di­sulfide bridges in HEWL and thaumatin at 100 K. Negative electron density around the sulfur sites suggested that the cysteines also sustained damage at room temperature, but alternative conformations were not found by *qFit* (Fig. 2 ▸).

**Figure 3:**
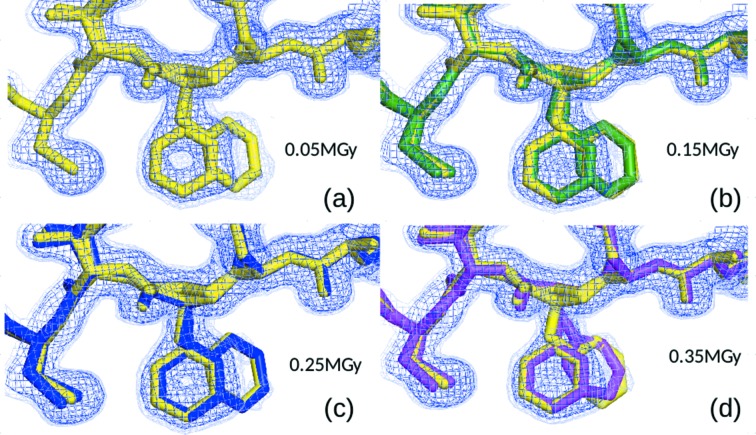
2*F*_o_ − *F*_c_ electron density maps showing the CypA F113 side chain contoured at 1σ (blue) and 0.3σ (light blue) for increasingly damaged data. The least damaged model (in yellow) is shown together with the more damaged models for reference. The DWD for each model was (*a*) 0.016 MGy, (*b*) 0.038 MGy, (*c*) 0.06 MGy and (*d*) 0.124 MGy. As radiation damage progresses, the electron density for the minor conformer becomes less defined and the refined position of the alternate side chain moves closer to the major conformer.

Residues implicated in enzymatic activity of CypA (F113, M61, S99 and the catalytic residue R55) showed only minor electron density changes as a function of absorbed dose (see Fig. 3 ▸), suggesting that radiation damage is not responsible for the appearance of distinct conformations.

**Figure 4:**
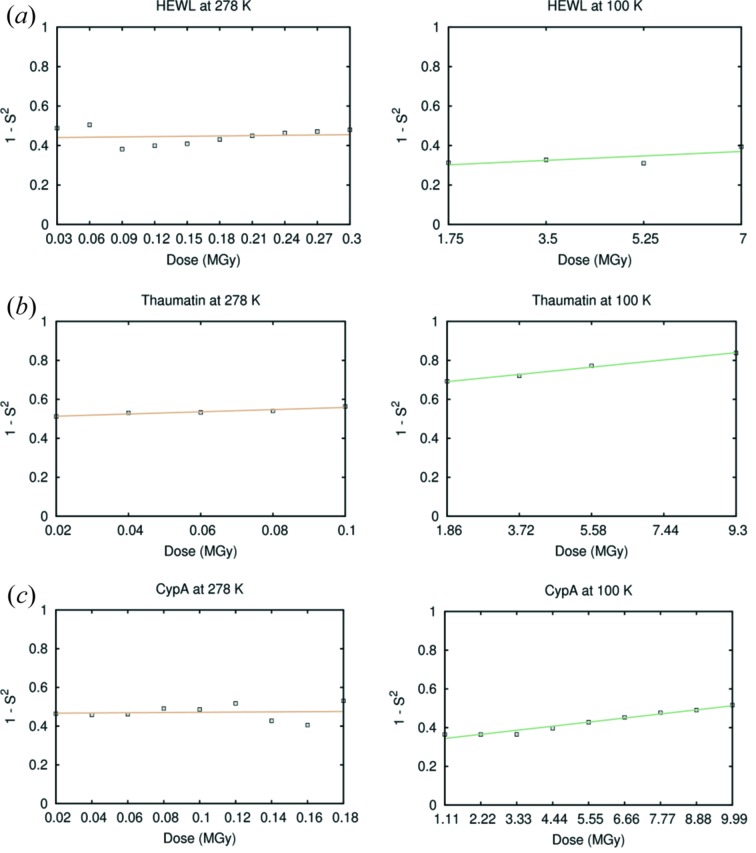
Average side-chain order parameters as a function of DWD for HEWL (*a*), thaumatin (*b*) and CypA (*c*) at 100 and 278 K.

We next used a measure of disorder in multiconformer models, the complement of the *B*-order parameter (1 − *S* ^2^), to track the effect of radiation damage on conformational disorder in each temperature regime (Fig. 4 ▸). Interestingly, this metric suggests a clear correlation between absorbed dose and conformational disorder at cryogenic temperature, but not at room temperature (Fig. 4 ▸). A linear fit of 1 − *S* ^2^, averaged over all residues, against dose indicates a highly significant increase in disorder at 100 K from lowest to highest radiation dose: around 20% for thaumatin (*p* < 0.005) and around 35% for CypA (*p* < 10^−6^) between the first and last dataset in the series. The total increase for HEWL is not significant (*p* = 0.256), although similar to that of thaumatin. At 278 K, the disorder increases by only 2% and 3%, for HEWL and CypA, respectively, and is not statistically significant (*p* = 0.740 and 0.846, respectively). The increase of 8% for thaumatin (*p* = 0.009) is statistically significant, but 2.5 times smaller than at 278 K. Surprisingly, the value at low dose for 1 − *S* ^2^ for thaumatin was higher at 100 K than at 278 K. At low dose, the data collected at 100 K also had a higher average *B*-factor (about 26 Å^2^ for the lowest dose dataset) than the data at 278 K (19 Å^2^), which is uncommon, but thaumatin cryo-structures in the Protein Data Bank (PDB) exhibit a wide range of values from 7.6 Å^2^ (PDB ID 4c3c) to 23.6 Å^2^ (PDB ID 3v88), indicating that the high value at 100 K could be due to sample-to-sample variation.

**Figure 5:**
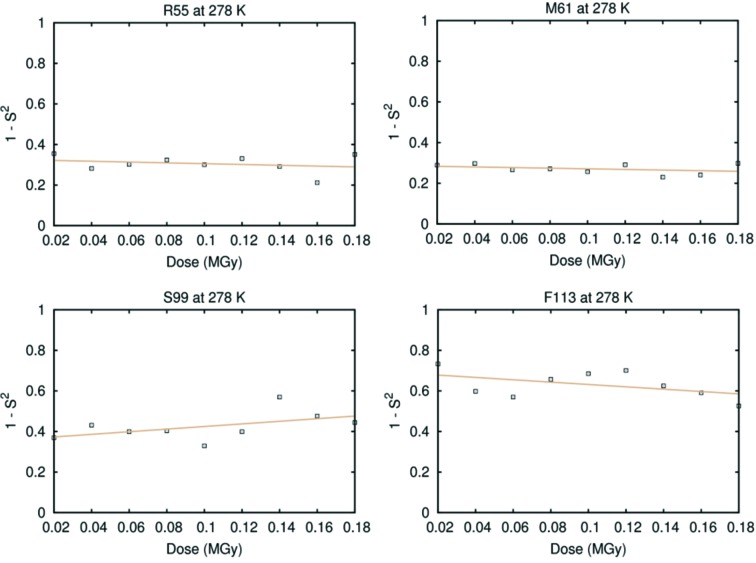
Order parameters as a function of DWD for the CypA residues R55 (slope = −0.199; *p* = 0.513), M61 (slope = −0.153; *p* = 0.373), S99 (slope = 0.643; *p* = 0.161) and F113 (slope = −0.579; *p* = 0.201) at 278 K.

Fig. 5 ▸ shows 1 − *S* ^2^ for the CypA side chains F113, M61, S99 and R55 at 278 K. None of these residues shows a statistically significant relationship between dose and disorder. The electron density maps do not show many changes as the dose increases, except for an overall reduction in the density level, as shown in Fig. 3 ▸, consistent with the global radiation damage and loss of resolution.

## 4. Discussion   

Automated software for modelling multiple conformers in low-level electron density has enabled detailed, unbiased analysis of the conformational ensemble of biological molecules, especially for data collected at room temperature. Here, the conformational ensembles of three proteins subjected to increasing doses of radiation were examined for signs of damage at 100 K and 278 K. We found that crystal structures respond differently to increasing dose at these temperatures. At both temperatures the conformational heterogeneity (in terms of the number of alternative conformations) of the crystal structures is at most minimally affected by dose. However, cryogenic crystals respond to dose with elevated conformational dynamics encoded by the multi-conformer order parameter up to DWDs close to 10 MGy, exceeding the disorder values measured for the room-temperature structures. Because this type of order parameter is based on alternative conformations and *B*-factors, these two facts together suggest that the dominant effects of radiation damage at cryogenic temperatures can be modelled by *B*-factors (Warkentin & Thorne, 2010 ▸).

By contrast, the order parameters of room-temperature crystals were generally unaffected by the much lower dose accumulated over the time of the experiment. The sequential radiation damage model of protein decay in X-rays at room temperature (Sygusch & Allaire, 1988 ▸; Southworth-Davies *et al.*, 2007 ▸) predicts that crystalline samples go through a partially disordered phase that contributes to diffraction to high angle before becoming totally dis­ordered. While this model predicts a significant increase in disorder during the phase transition, our analysis has not found clear evidence of this process. This discrepancy may be due to the difference between the ordered and partially disordered fractions being too subtle to be modelled using our approaches. Indeed, the conformational changes may be too subtle to detect in the electron density with existing tools, such as *qFit* or *Ringer* (Lang *et al.*, 2014 ▸). Alternatively, the dose used in our study may not be high enough to detect the intermediate state; nevertheless, it was sufficient to determine high-resolution room-temperature structures. Our findings are also consistent with a fast transition from ordered to totally disordered structure, with only the more ordered fraction contributing to the electron density.

We confirmed that, despite the faster decay of crystals at room temperature, many sites where damage is concentrated at 100 K do not appear affected to the same extent at room temperature. Although the comparison between datasets shows evidence of specific radiation damage, the progression is not the same in the two temperature regimes: the most notable effects at 100 K (de­carboxyl­ation of acidic side chains and reduction of di­sulfide bridges) are less prominent at 278 K. This difference could explain the results of Roedig *et al.* (2016 ▸), who found no visible specific damage at room temperature for doses up to 0.5 MGy, which is of the same order of magnitude as the doses used at 278 K in this study. The difference in response by specific sites to radiation between the two temperature regimes could be an effect of the much smaller dose received by the crystals at room temperature being deposited over a larger number of susceptible sites: the crystal is already destroyed by the time some changes would be clearly detectable. However, the amount of damage to a specific site may be different at different dose rates. If this is the case, data collection at high dose rates to outrun radiation damage at room temperature (Owen *et al.*, 2012 ▸) might counter-intuitively result in more extensive specific damage affecting the structure. Also, at temperatures slightly above the glass transition, where more radical species are mobile but the crystal will tolerate a larger dose (Warkentin *et al.*, 2013 ▸), the damage to specific sites over the course of the experiment could be more visible. Structures at these intermediate temperatures are likely to be similar to the ones at room temperature, making this intermediate temperature regime interesting for future experiments to examine the temperature-dependent effects of radiation damage.

## 5. Conclusions   

Our results signify that there is no relationship between specific radiation damage at side chains and large shifts in their conformations at 278 and 100 K, except for the case of broken di­sulfide bridges at 100 K. Only weak correlations were found between disorder and dose at 278 K over typical doses used for experiments at room temperature. Therefore, it seems unlikely that radiation damage is responsible for the alternate conformations responsible for biological activity in, for example, CypA. These findings were consistent for the three proteins studied, suggesting that conformational dynamics at room temperature is generally unaffected by radiation damage, if the experiment is carefully designed. However, at sufficiently high dose, radiation damage could mask minor conformers due to overall loss of electron density. Moreover, although specific damage to key residues was not detected here, it cannot be ruled out in the general case. As long as experiments at X-ray free-electron laser sources are not commonplace, careful monitoring of experiments based on the detection of subtle conformational changes will be a necessity.

The approach used here, using multiple crystals to collect increasingly damaged data with multi-conformer *qFit* models as readout, can be useful to monitor trends and effects of radiation damage on experiments at different temperatures and conditions. Although the analysis methods we used are limited to samples that diffract to high resolution and well ordered areas of the protein, any consistent findings could be of general use.

## Supplementary Material

Supplementary tables listing refinement statistics and difference map peak analysis. DOI: 10.1107/S1600577516017343/gm5050sup1.pdf

## Figures and Tables

**Table:** At 278 K the values are calculated after merging the data from all the crystals listed in Table S1 of the supporting information. The resolution given indicates the value to which data were scaled and merged. The actual data resolution varies between datasets and it typically increases between 0.1 and 0.2 Å between the least and most damaged datasets, except for HEWL 100 K data, where the actual resolution limit is beyond the detector edge for all datasets and could not be determined.

HEWL 278 K					
Dataset #	1	2	3	4	5
Resolution (Å)†	35.4–1.20 (1.26–1.20)	35.39–1.20 (1.26–1.20)	35.39–1.20 (1.26–1.20)	35.39–1.20 (1.26–1.20)	35.40–1.20 (1.26–1.20)
*R*_merge_ †	0.107 (0.443)	0.109 (0.519)	0.110 (0.609)	0.113 (0.736)	0.113 (0.941)
〈*I*〉/〈σ*I*〉†	10.6 (2.5)	10.0 (2.2)	9.8 (1.8)	9.3 (1.5)	8.8 (1.2)
Completeness (%)†	93.9 (86.9)	93.9 (86.8)	93.9 (86.7)	93.9 (86.8)	93.9 (86.7)
Multiplicity†	3.5 (3.1)	3.5 (3.1)	3.5 (3.1)	3.5 (3.0)	3.5 (3.0)

Dataset #	6	7	8	9	10
Resolution (Å)†	35.4–1.20 (1.26–1.20)	35.40–1.20 (1.26–1.20)	35.40–1.20 (1.26–1.20)	35.40–1.20 (1.26–1.20)	35.4–1.20 (1.26–1.20)
*R*_merge_ †	0.117 (1.234)	0.120 (1.640)	0.123 (2.113)	0.126 (2.638)	0.134 (4.035)
〈*I*〉/〈σ*I*〉†	8.2 (1.0)	7.8 (0.8)	7.3 (0.6)	6.7 (0.5)	6.1 (0.4)
Completeness (%)†	93.9 (86.9)	93.9 (86.9)	93.7 (85.9)	93.8 (86.5)	93.7 (85.8)
Multiplicity†	3.5 (3.0)	3.5 (3.0)	3.5 (3.0)	3.5 (3.0)	3.5 (3.0)

**Table:** 

HEWL 100 K				
Dataset #	1	2	3	4
Resolution (Å)†	35.65–1.20 (1.26–1.20)	38.66–1.20 (1.26–1.20)	38.65–1.20 (1.26–1.20)	38.67–1.20 (1.26–1.20)
*R*_merge_ †	0.025 (0.098)	0.025 (0.118)	0.025 (0.098)	0.032 (0.240)
〈*I*〉/〈σ*I*〉†	45.8 (15.0)	38.6 (11.3)	45.8 (15.0)	32.8 (6.8)
Completeness (%)†	99.6 (97.7)	99.5 (97.2)	99.6 (97.7)	99.6 (97.9)
Multiplicity†	6.6 (5.8)	5.2 (4.6)	6.6 (5.8)	6.6 (5.8)

**Table:** 

Thaumatin 278 K					
Dataset #	1	2	3	4	5
Resolution (Å)†	37.89–1.55 (1.63–1.55)	37.89–1.55 (1.63–1.55)	37.89–1.55 (1.63–1.55)	37.89–1.55 (1.63–1.55)	37.89–1.55 (1.63–1.55)
*R*_merge_ †	0.093 (0.682)	0.094 (0.713)	0.096 (0.826)	0.096 (0.840)	0.096 (0.963)
〈*I*〉/〈σ*I*〉†	6.8 (0.9)	6.7 (0.9)	6.5 (0.8)	6.4 (0.7)	6.3 (0.6)
Completeness (%)†	82.1 (42.5)	82.0 (42.6)	82.0 (42.5)	82.0 (42.4)	82.1 (42.5)
Multiplicity†	2.3 (1.3)	2.2 (1.3)	2.2 (1.3)	2.3 (1.3)	2.3 (1.3)

**Table:** 

Thaumatin 100 K				
Dataset #	1	2	3	4
Resolution (Å)†	37.85–1.59 (1.68–1.59)	37.91–1.59 (1.68–1.59)	37.95–1.59 (1.68–1.59)	37.99–1.59 (1.68–1.59)
*R*_merge_ †	0.044 (0.728)	0.045 (1.026)	0.049 (1.540)	0.059 (3.129)
〈*I*〉/〈σ*I*〉†	15.1 (1.6)	14.7 (1.2)	13.5 (0.8)	11.1 (0.4)
Completeness (%)†	99.0 (95.3)	99.0 (94.8)	99.1 (95.2)	99.0 (94.5)
Multiplicity†	4.1 (3.4)	4.1 (3.4)	4.1 (3.8)	4.1 (3.3)

**Table:** 

CypA 278 K					
Dataset #	1	2	3	4	5
Resolution (Å)†	38.68–1.49 (1.52–1.49)	38.67–1.49 (1.52–1.49)	38.66–1.49 (1.52–1.49)	38.67–1.49 (1.52–1.49)	38.70–1.49 (1.52–1.49)
*R*_merge_ †	0.109 (0.526)	0.108 (0.640)	0.118 (0.896)	0.139 (1.470)	0.139 (1.652)
〈*I*〉/〈σ*I*〉†	18 (1.9)	12.9 (2.0)	16.1 (1.5)	15.9 (1.7)	24.5 (4.0)
Completeness (%)†	85.9 (65.1)	85.9 (65.1)	85.5 (64.5)	85.4 (64.2)	85.4 (64.1)
Multiplicity†	2.8 (2.3)	2.9 (2.3)	2.8 (2.3)	2.8 (2.2)	2.8 (2.2)

Dataset #	6	7	8	9	
Resolution (Å)†	38.70–1.49 (1.52–1.49)	38.71–1.49 (1.52–1.49)	38.70–1.49 (1.52–1.49)	38.74–1.49 (1.52–1.49)	
*R*_merge_ †	0.163 (2.299)	0.188 (2.484)	0.233 (2.892)	0.324 (2.795)	
〈*I*〉/〈σ*I*〉†	18.8 (2.1)	11.6 (0.8)	14.1 (0.8)	11.0 (0.8)	
Completeness (%)†	85.4 (64.3)	85.5 (64.1)	85.5 (64.3)	85.3 (63.5)	
Multiplicity†	2.8 (2.2)	2.8 (2.2)	2.8 (2.2)	2.8 (2.2)	

**Table:** 

CypA 100 K					
Dataset #	1	2	3	4	5
Resolution (Å)†	38.27–1.48 (1.51–1.48)	38.27–1.48 (1.51–1.48)	38.26–1.48 (1.51–1.48)	38.26–1.48 (1.51–1.48)	38.24–1.48 (1.51–1.48)
*R*_merge_ †	0.055 (0.525)	0.056 (0.572)	0.054 (0.630)	0.055 (0.719)	0.058 (0.827)
〈*I*〉/〈σ*I*〉†	13.1 (1.5)	14.8 (1.7)	13.3 (1.3)	12.9 (1.3)	12.8 (1.0)
Completeness (%)†	93.7 (55.3)	93.5 (51.1)	93.4 (49.3)	93.6 (52.1)	94.0 (57.3)
Multiplicity†	2.8 (2.2)	2.8 (2.2)	2.8 (2.2)	2.8 (2.2)	2.8 (2.2)

Dataset #	6	7	8	9	
Resolution (Å)†	38.24–1.48 (1.51–1.48)	38.23–1.48 (1.51–1.48)	38.24–1.48 (1.51–1.48)	38.23–1.48 (1.51–1.48)	
*R*_merge_ †	0.058 (0.957)	0.061 (1.030)	0.062 (1.182)	0.063 (1.398)	
〈*I*〉/〈σ*I*〉†	10.7 (0.9)	13.3 (1.2)	12.3 (1.1)	14.5 (1.1)	
Completeness (%)†	93.9 (57.3)	94.0 (58.2)	94.0 (58.6)	93.7 (54.1)	
Multiplicity†	2.8 (2.2)	2.8 (2.2)	2.8 (2.2)	2.8 (2.2)	

† Outermost resolution shell values are in parentheses.

**Table 2:** Number of total alternate conformations and distinct alternate conformations (different rotamers) as a function of DWD for HEWL (129 residues), thaumatin (207 residues) and CypA (164 residues) at 278 and 100 K The slope for a least-squares linear fit (number of residues in distinct conformations/MGy) and the *p* value are given for each protein and temperature.

Protein	Temperature (K)	DWD (MGy)	Total # conformers	Distinct #
HEWL	278	0.03	78	11
		0.06	75	16
		0.08	75	17
		0.11	74	14
		0.14	54	12
		0.17	71	13
		0.20	55	12
		0.22	57	10
		0.25	64	10
		0.28	69	18
Slope = −4.12	*p* = 0.74			
	100	1.75	85	23
		3.50	90	24
		5.25	91	23
		7.00	93	29
Slope = 0.97	*p* = 0.235			

Thaumatin	278	0.02	117	28
		0.05	109	27
		0.07	126	28
		0.09	114	26
		0.12	96	27
Slope = −12.07	*p* = 0.337			
	100	1.86	94	22
		3.72	93	13
		5.58	95	26
		9.30	87	30
Slope = 1.58	*p* = 0.309			

CypA	278	0.02	84	26
		0.03	78	25
		0.05	84	28
		0.06	89	29
		0.08	85	29
		0.10	88	28
		0.11	84	31
		0.13	76	30
		0.14	76	20
Slope = −2.70	*p* = 0.923			
	100	1.11	73	24
		2.22	66	21
		3.33	73	21
		4.44	70	18
		5.55	69	21
		6.66	63	19
		7.77	66	30
		8.88	69	23
		9.99	65	24
Slope = 0.38	*p* = 0.397			
