# Supplementary material for: Conformational variation of proteins at room temperature is not dominated by radiation damage
Source: J Synchrotron Radiat. 2017 Jan 1;24(Pt 1):73–82. doi: 10.1107/S1600577516017343 (PMC5182021; doi:10.1107/S1600577516017343)
Supplement: Supplementary file 1 [file s-24-00073-sup1.pdf]

JOURNAL OF SYNCHROTRON RADIATION

**Volume 24 (2017)**

**Supporting information for article:**

# Conformational variation of proteins at room temperature is not dominated by radiation damage

**Silvia Russi, Ana González, Lillian R. Kenner, Daniel A. Keedy, James S. Fraser and Henry van den Bedem**

**Table S1** Data collection parameters.

| **Sample** | **Detector distance (mm)** | **Images/dataset** | **Δphi (degrees)** | **Diffraction-weighted dose (DWD)/dataset (MGy)** | **Number of datasets** |
|---|---|---|---|---|---|
| **HEWL A (100 K)** | 95.0 | 720 | 0.5 | 1.75 | 4 |
| **HEWL A (278 K)** | 98.8 | 6 | 1 | 0.03 | 10 |
| **HEWL B (278 K)** | 95.0 | 16 | 0.65 | 0.03 | 10 |
| **HEWL C (278 K)** | 95.0 | 9 | 0.8 | 0.03 | 10 |
| **HEWL D (278 K)** | 95.0 | 8 | 0.75 | 0.03 | 10 |
| **HEWL E (278 K)** | 95.0 | 9 | 0.6 | 0.03 | 10 |
| **HEWL F (278 K)** | 95.0 | 9 | 0.6 | 0.03 | 10 |
| | | | | | |
| **Thaumatin A (100 K)** | 180.0 | 185 | 0.5 | 1.86 | 4* |
| **Thaumatin A (278 K)** | 187.6 | 5 | 1 | 0.02 | 5 |
| **Thaumatin B (278 K)** | 172.9 | 5 | 1 | 0.02 | 5 |
| **Thaumatin C (278 K)** | 172.9 | 5 | 0.9 | 0.02 | 5 |
| **Thaumatin D (278 K)** | 187.7 | 4 | 1 | 0.02 | 5 |
| **Thaumatin E (278 K)** | 172.9 | 7 | 0.6 | 0.02 | 5 |

| | | | | | |
|---|---|---|---|---|---|
| **Thaumatin F (278 K)** | 156.0 | 5 | 0.85 | 0.02 | 5 |
| **Thaumatin G (278 K)** | 172.9 | 5 | 0.8 | 0.02 | 5 |
| **Thaumatin H (278 K)** | 180.0 | 4 | 1 | 0.02 | 5 |
| *Five data sets were collected from thaumatin at 100 K, but the next to last was not used because it caused the conformation analysis to fail, for reasons unknown. | | | | | |
| **CypA A (100 K)** | 175.0 | 591 | 0.6 | 1.11 | 9 |
| **CypA A (278 K)** | 200.0 | 10 | 0.45 | 0.02 | 9 |
| **CypA B (278 K)** | 200.0 | 10 | 0.45 | 0.02 | 9 |
| **CypA C (278 K)** | 200.0 | 10 | 0.45 | 0.02 | 9 |
| **CypA D (278 K)** | 206.8 | 42 | 0.2 | 0.02 | 9 |
| **CypA E (278 K)** | 200.0 | 10 | 0.2 | 0.02 | 9 |
| **CypA F (278 K)** | 200.0 | 10 | 0.2 | 0.02 | 9 |
| **CypA G (278 K)** | 200.0 | 14 | 0.5 | 0.02 | 9 |
| **CypA H (278 K)** | 200.0 | 14 | 0.5 | 0.02 | 9 |
| **CypA I (278 K)** | 200.0 | 14 | 0.5 | 0.02 | 9 |
| **CypA J (278 K)** | 200.0 | 8 | 0.45 | 0.02 | 9 |
| **CypA K (278 K)** | 200.0 | 10 | 0.45 | 0.02 | 9 |

**Table S2** Structure refinement results. There is one molecule per asymmetric unit in all cases.

| HEWL 278 K | | | | | |
|---|---|---|---|---|---|
| **Dataset #** | **1** | **2** | **3** | **4** | **5** |
| **Resolution (Å)** | 1.20 | 1.20 | 1.20 | 1.20 | 1.20 |
| **No. reflections** | 34048 | 33044 | 34021 | 33992 | 33832 |
| **R work/R free** | 0.161/ 0.192 | 0.162/ 0.196 | 0.164/ 0.187 | 0.168/ 0.193 | 0.175/ 0.206 |
| **No. atoms in asymmetric unit** | | | | | |
| **Protein** | 1851 | 1882 | 1792 | 1855 | 1455 |
| **Solvent** | 82 | 85 | 73 | 64 | 58 |
| **R.M.S. deviations** | | | | | |
| **Bond lengths (Å)** | 0.024 | 0.025 | 0.025 | 0.024 | 0.026 |
| **Bond angles (°)** | 2.441 | 2.505 | 2.355 | 2.353 | 2.441 |
| **Dataset #** | **6** | **7** | **8** | **9** | **10** |
| **Resolution (Å)** | 1.20 | 1.20 | 1.20 | 1.20 | 1.20 |
| **No. reflections** | 33678 | 33066 | 32587 | 31769 | 30295 |
| **R work/R free** | 0.176/ 0.204 | 0.178/ 0.207 | 0.181/ 0.210 | 0.182/ 0.211 | 0.182/ 0.218 |
| **No. atoms in asymmetric unit** | | | | | |
| **Protein** | 1562 | 1586 | 1638 | 1649 | 1787 |
| **Solvent** | 67 | 58 | 52 | 50 | 57 |
| **R.M.S. deviations** | | | | | |
| **Bond lengths (Å)** | 0.023 | 0.022 | 0.022 | 0.022 | 0.019 |
| **Bond angles (°)** | 2.299 | 2.113 | 2.267 | 2.187 | 2.031 |

| **HEWL 100 K** | | | | |
|---|---|---|---|---|
| **Dataset #** | **1** | **2** | **3** | **4** |
| **Resolution (Å)** | 1.20 | 1.20 | 1.20 | 1.20 |
| **No. reflections** | 35966 | 35993 | 35996 | 35967 |
| **R work/R free** | 0.150/ 0.180 | 0.150/ 0.164 | 0.152/ 0.180 | 0.159/ 0.178 |
| **No. atoms in asymmetric unit** | | | | |
| **Protein** | 2009 | 2075 | 1975 | 2120 |
| **Solvent** | 160 | 148 | 157 | 123 |
| **R.M.S. deviations** | | | | |
| **Bond lengths (Å)** | 0.010 | 0.014 | 0.012 | 0.025 |
| **Bond angles (°)** | 1.356 | 1.442 | 1.514 | 1.650 |

| **Thaumatin 278 K** | | | | | |
|---|---|---|---|---|---|
| **Dataset #** | **1** | **2** | **3** | **4** | **5** |
| **Resolution (Å)** | 1.55 | 1.55 | 1.55 | 1.55 | 1.55 |
| **No. reflections** | 30297 | 30229 | 30217 | 30204 | 30188 |
| **R work/R free** | 0.143/ 0.179 | 0.145/ 0.181 | 0.146/ 0.185 | 0.148/ 0.186 | 0.150/ 0.190 |
| **No. atoms in asymmetric unit** | | | | | |
| **Protein** | 2535 | 2368 | 2529 | 2442 | 2227 |
| **Solvent** | 151 | 153 | 156 | 149 | 153 |
| **R.M.S. deviations** | | | | | |
| **Bond lengths (Å)** | 0.019 | 0.020 | 0.019 | 0.019 | 0.019 |
| **Bond angles (°)** | 1.988 | 1.950 | 2.029 | 1.978 | 1.934 |

| **Dataset #** | **1** | **2** | **3** | **4** |
|---|---|---|---|---|
| **Resolution (Å)** | 1.59 | 1.59 | 1.59 | 1.59 |
| **No. reflections** | 32840 | 32906 | 32791 | 30337 |
| **R work/R free** | 0.162/ 0.196 | 0.166/ 0.191 | 0.169/ 0.204 | 0.173/ 0.212 |
| **No. atoms in asymmetric unit** | | | | |
| **Protein** | 2344 | 2359 | 2355 | 2270 |
| **Solvent** | 174 | 169 | 141 | 118 |
| **R.M.S. deviations** | | | | |
| **Bond lengths (Å)** | 0.021 | 0.021 | 0.020 | 0.019 |
| **Bond angles (°)** | 2.150 | 2.080 | 2.006 | 2.012 |

| **CypA 278 K** | | | | | |
|---|---|---|---|---|---|
| **Dataset #** | **1** | **2** | **3** | **4** | **5** |
| **Resolution (Å)** | 1.7 | 1.7 | 1.7 | 1.7 | 1.7 |
| **No. reflections** | 20749 | 20713 | 20589 | 20527 | 20508 |
| **R work/R free** | 0.153/ 0.184 | 0.155/ 0.193 | 0.156/ 0.198 | 0.155/ 0.203 | 0.155/ 0.201 |
| **No. atoms in asymmetric unit** | | | | | |
| **Protein** | 1850 | 1819 | 1877 | 1921 | 1883 |
| **Solvent** | 127 | 129 | 129 | 129 | 129 |
| **R.M.S. deviations** | | | | | |
| **Bond lengths (Å)** | 0.006 | 0.006 | 0.006 | 0.006 | 0.006 |
| **Bond angles (°)** | 0.784 | 0.812 | 0.808 | 0.789 | 0.811 |
| **Dataset #** | **6** | **7** | **8** | **9** | |
| **Resolution (Å)** | 1.70 | 1.70 | 1.70 | 1.70 | |
| **No. reflections** | 20222 | 20245 | 19950 | 19706 | |

| | | | | | |
|---|---|---|---|---|---|
| **R work/R free** | 0.160/<br>0.200 | 0.164/<br>0.210 | 0.169/<br>0.224 | 0.174/<br>0.223 | |
| **No. atoms in asymmetric unit** | | | | | |
| **Protein** | 1904 | 1888 | 1771 | 1826 | |
| **Solvent** | 128 | 129 | 129 | 127 | |
| **R.M.S. deviations** | | | | | |
| **Bond lengths (Å)** | 0.006 | 0.006 | 0.007 | 0.006 | |
| **Bond angles (°)** | 0.819 | 0.820 | 0.818 | 0.856 | |

| **CypA 100 K** | | | | | |
|---|---|---|---|---|---|
| **Dataset #** | **1** | **2** | **3** | **4** | **5** |
| **Resolution (Å)** | 1.70 | 1.70 | 1.70 | 1.70 | 1.70 |
| **No. reflections** | 21705 | 21735 | 21779 | 21797 | 21741 |
| **R work/R free** | 0.181/<br>0.219 | 0.174/<br>0.208 | 0.176/<br>0.214 | 0.170/<br>0.211 | 0.172/<br>0.207 |
| **No. atoms in asymmetric unit** | | | | | |
| **Protein** | 1758 | 1787 | 1793 | 1783 | 1781 |
| **Solvent** | 269 | 253 | 256 | 244 | 249 |
| **R.M.S. deviations** | | | | | |
| **Bond lengths (Å)** | 0.007 | 0.006 | 0.006 | 0.006 | 0.006 |
| **Bond angles (°)** | 1.064 | 0.803 | 0.772 | 0.767 | 0.800 |
| **Dataset #** | **6** | **7** | **8** | **9** | |
| **Resolution (Å)** | 1.70 | 1.70 | 1.70 | 1.70 | |
| **No. reflections** | 21691 | 21632 | 21687 | 21655 | |
| **R work/R free** | 0.172/<br>0.213 | 0.173/<br>0.208 | 0.171/<br>0.194 | 0.174/<br>0.213 | |
| **No. atoms in asymmetric unit** | | | | | |

| | | | | | |
|---|---|---|---|---|---|
| **Protein** | 1728 | 1783 | 1744 | 1767 | |
| **Solvent** | 242 | 247 | 230 | 232 | |
| **R.M.S. deviations** | | | | | |
| **Bond lengths (Å)** | 0.006 | 0.006 | 0.006 | 0.006 | |
| **Bond angles (°)** | 0.812 | 0.784 | 0.785 | 0.810 | |

**Table S3** List of residues with different side chain rotamers in the first (lowest dose) and last (highest dose) models. The average B-factor for the side chain and the accessible surface area (ASA) for each listed residue in the first model were calculated with the programs BAVERAGE (Winn *et al*., 2011) and AREAIMOL (Lee & Richards, 1971) respectively. The $1\text{-}S^2$ values for the first model are also listed. The peak height in the difference Fo-Fo map is given as a multiple of the map RMS.

| **HEWL 278 K** | | | | |
|---|---|---|---|---|
| **Residue** | **Peak height** | **ASA (Å$^2$)** | **<B> (Å$^2$)** | **1-S$^2$** |
| R5 | 3.90 (324) | 72.0 | 20.025 | 0.527 |
| D18 | 3.86 (330) | 55.3 | 23.111 | 0.775 |
| S24 | 5.03 (164) | 24.2 | 13.327 | 0.521 |
| R45 | 4.41 (244) | 139.8 | 19.145 | 0.642 |
| Y53 | 6.60 (37) | 20.0 | 9.289 | 0.265 |
| R73 | 3.70 (355) | 243.0 | 25.918 | 0.817 |
| N77 | 3.22 (437) | 156.8 | 20.850 | 0.659 |
| I78 | 3.37 (417) | 32.3 | 15.891 | 0.591 |
| D87 | 4.00 (307) | 96.2 | 19.038 | 0.632 |
| N93 | 4.64 (217) | 71.8 | 18.113 | 0.878 |
| D101 | 2.69 (-) | 82.2 | 23.237 | 0.642 |
| V109 | 4.22 (269) | 77.7 | 13.812 | 0.884 |
| D119 | 1.59 (-) | 102.3 | 19.889 | 0.647 |

| | | | | |
|---|---|---|---|---|
| Q121 | 5.95 (78) | 58.3 | 30.929 | 0.849 |
| I124 | 3.13 (456) | 41.2 | 18.174 | 0.579 |
| R125 | 3.08 (483) | 236.2 | 25.175 | 0.940 |

| **HEWL 100 K** | | | | |
|---|---|---|---|---|
| **Residue** | **Peak height** | **ASA (Å$^2$)** | **<B> (Å$^2$)** | **1-S$^2$** |
| L17 | 3.11 (-) | 0.0 | 11.661 | 0.634 |
| *Y*20 | 4.73 (203) | 70.0 | 10.840 | 0.282 |
| R21 | 3.16 (-) | 170.7 | 16.752 | 0.323 |
| C30 | 25.60 (1) | 0.5 | 7.940 | 0.198 |
| T47 | 4.22 (297) | 168.7 | 14.304 | 0.790 |
| N59 | 6.8 (41) | 16.3 | 10.268 | 0.266 |
| C94 | 23.1 (3) | 1.0 | 9.271 | 0.231 |
| R112 | 4.39 (264) | 119.3 | 12.898 | 0.26 |

| **Thaumatin 278 K** | | | | |
|---|---|---|---|---|
| **Residue** | **Peak height** | **ASA (Å$^2$)** | **<B> (Å$^2$)** | **1-S$^2$** |
| E42 | 3.6 (288) | 149.2 | 38.418 | 0.816 |
| D55 | 5.4 (19) | 80.8 | 26.514 | 0.538 |
| K78 | 3.0 (-) | 129.0 | 33.776 | 0.941 |
| R82 | 3.0 (-) | 184.2 | 24.220 | 0.578 |
| K139 | 4.0 (152) | 165.8 | 28.440 | 0.611 |
| R171 | 3.6 (288) | 98.7 | 17.570 | 0.388 |

| **Thaumatin 100 K** | | | | |
|---|---|---|---|---|
| **Residue** | **Peak height** | **ASA (Å$^2$)** | **<B> (Å$^2$)** | **1-S$^2$** |
| T2 | 2.8 (-) | 48.3 | 38.653 | 0.833 |
| R8 | 5.8 (64) | 125.7 | 22.752 | 0.567 |
| S10 | 5.1 (131) | 114.8 | 22.420 | 0.775 |
| A23 | 1.9 (-) | 37.0 | 25.310 | 0.804 |
| N40 | 3.0 (-) | 91.3 | 54.765 | 1 |
| E42 | 2.7 (-) | 169.7 | 40.975 | 0.925 |
| K49 | 4.1 (306) | 43.5 | 23.429 | 0.544 |
| K67 | 4.1 (306) | 115.5 | 33.417 | 0.778 |
| R76 | 2.3 (-) | 180.5 | 42.316 | 0.882 |
| K78 | 2.7 (-) | 112.0 | 41.367 | 0.930 |
| R79 | 1.9 (-) | 174.3 | 44.614 | 0.901 |
| R82 | 3.9 (360) | 210.3 | 27.667 | 0.591 |
| Q94 | 4.0 (327) | 35.2 | 30.386 | 0.765 |
| K106 | 3.3 (-) | 91.7 | 25.792 | 0.587 |
| K139 | 3.9 (360) | 124.8 | 32.220 | 0.877 |
| C159 | 6.8 (33) | 13.8 | 25.513 | 0.884 |

| **CypA 278 K** | | | | |
|---|---|---|---|---|
| **Residue** | **Peak height** | **ASA (Å$^2$)** | **<B> (Å$^2$)** | **1-S$^2$** |
| V6 | 3.6 (-) | 0 | 15.0 | 0.698 |
| E15 | 2.0 (-) | 122.3 | 52.7 | 0.586 |
| V20 | 4.0 (444) | 1.2 | 11.5 | 0.310 |

| | | | | |
|---|---|---|---|---|
| S21 | 4.0 (444) | 1.3 | 20.1 | 0.568 |
| F22 | 4.1 (418) | 0.3 | 12.7 | 0.398 |
| F36 | 4.9 (260) | 0.2 | 12.0 | 0.306 |
| S40 | 3.8 (477) | 2.5 | 12.7 | 0.597 |
| K44 | 2.8 (-) | 139.2 | 32.7 | 0.845 |
| F46 | 3.3 (-) | 51.5 | 25.1 | 0.625 |
| I56 | 4.1 (418) | 0.0 | 12.5 | 0.214 |
| F67 | 4.6 (305) | 33.7 | 12.5 | 0.296 |
| M100 | 10.4 (3) | 0.0 | 13.1 | 0.351 |
| T116 | 5.6 (145) | 19.7 | 15.4 | 0.503 |
| K118 | 3.3 (-) | 111.7 | 37.6 | 0.531 |
| K131 | 2.7 (-) | 96.0 | 29.2 | 0.564 |
| M136 | 6.8 (30) | 45.5 | 21.8 | 0.486 |
| R144 | 1.7 (-) | 240.2 | 41.5 | 0.727 |
| R148 | 3.3 (-) | 218.8 | 29.0 | 0.552 |
| K151 | 2.7 (-) | 210.2 | 32.7 | 0.846 |
| K155 | 2.1 (-) | 134.2 | 34.7 | 0.734 |

| **CypA 100K** | | | | |
|---|---|---|---|---|
| **Residue** | **Peak height** | **ASA (Å$^2$)** | **<B> (Å$^2$)** | **1-S$^2$** |
| D27 | 5.3 (228) | 114.2 | 19.676 | 0.468 |
| R37 | 4.4 (431) | 46.8 | 16.738 | 0.273 |
| M61 | 6.9 (39) | 9.3 | 30.215 | 0.270 |
| T68 | 5.6 (173) | 72.7 | 12.008 | 0.462 |

| | | | | |
|---|---|---|---|---|
| K76 | 4.0 (-) | 82.0 | 15.432 | 0.559 |
| K91 | 3.8 (-) | 139.2 | 18.831 | 0.343 |
| T107 | 7.7 (16) | 24.2 | 12.830 | 0.318 |
| E120 | 3.5 (-) | 166.5 | 28.082 | 0.781 |
| K131 | 2.2 (-) | 110.2 | 23.892 | 0.841 |
| K133 | 1.5 (-) | 109.5 | 38.804 | 0.533 |
| E134 | 6.1 (96) | 135.7 | 18.893 | 0.859 |
| R144 | 4.3 (447) | 181.5 | 24.056 | 0.275 |
| K154 | 2.9 (-) | 122.7 | 18.727 | 0.475 |
